# Evaluating the Efficacy of MamaLift Plus Digital Therapeutic Mobile App for Postpartum Depression (SuMMER): Randomized, Placebo-Controlled Pivotal Trial

**DOI:** 10.2196/69050

**Published:** 2025-07-01

**Authors:** Shailja Dixit, Indira Malladi, Sidhartha Shankar, Amrik Shah

**Affiliations:** 1 Curio Digital Therapeutics Princeton, NJ United States

**Keywords:** postpartum depression, digital therapeutics, cognitive behavioral therapy, behavioral activation therapy, interpersonal therapy, dialectical behavioral therapy, mobile health, randomized controlled trial, Edinburgh Postnatal Depression Scale, MamaLift Plus

## Abstract

**Background:**

Improvement in the Edinburgh Postnatal Depression Scale (EPDS) score is a regulatory-approved measure for symptom improvement in postpartum depression (PPD). While digital solutions have the potential to overcome common treatment barriers, few have shown clinically significant improvement in PPD symptoms, as measured by the EPDS.

**Objective:**

This study aimed to evaluate the clinical efficacy of the MamaLift Plus digital therapeutic for the improvement of PPD symptoms in women who had recently given birth and had PPD, as assessed by the EPDS.

**Methods:**

This double-blind, randomized, placebo-controlled phase 3 pivotal trial recruited participants remotely. Eligibility criteria required that participants have an EPDS score between 13 and 19 and a confirmed diagnosis of PPD. Participants were randomized to the MamaLift Plus intervention or sham control arm, with stratification based on new mom status. “New moms” are those who have had one live birth. MamaLift Plus is a self-guided 8-week digital therapeutic for symptomatic treatment for PPD. MamaLift Plus can be used on a mobile device. MamaLift Plus delivers digital Cognitive Behavioral Therapy, Behavioral Activation Therapy, Interpersonal Therapy, and Dialectical Behavior Therapy for PPD. The sham control mimicked the features, functionality, and user experience of the treatment. The most important difference between the 2 arms was that participants in the sham control app did not receive any Cognitive Behavioral Therapy. Primary and secondary endpoints were self-assessed. The primary endpoint was the proportion of participants whose EPDS scores improved by ≥4 points at the end of the study assessment. The intent-to-treat (ITT) analysis set included all randomized participants who started at least 1 module. The full analysis set (FAS) population included all participants who completed at least one postbaseline assessment. The trial is closed.

**Results:**

Participants were recruited remotely between April 18 and May 24, 2023. Eligible participants were assessed by a licensed mental health provider to confirm a diagnosis of PPD. In addition, 95 participants were randomized to the intervention and 46 to the control groups. A total of 86.3% (82/95) of MamaLift Plus arm participants achieved an improvement of ≥4 points, compared with 23.9% (11/46) of sham control arm participants (*P*<.0001). There were 2 adverse events each in the intervention arm 2.1% (2/95) and sham control arm 4.3% (2/46). Only 11 participants failed to provide any postbaseline assessment for the primary endpoint.

**Conclusions:**

Participants who received MamaLift Plus exhibited significant and clinically meaningful improvement in depressive symptoms compared with control. Results suggest MamaLift Plus has the potential to improve treatment outcomes for women experiencing PPD.

**Trial Registration:**

ClinicalTrials.gov NCT05958095; https://clinicaltrials.gov/study/NCT05958095

## Introduction

### Background

Perinatal depression affects 1 in 7 women and is one of the most common and underdiagnosed obstetric complications [1]. According to the American College of Obstetricians and Gynecologists, depression onset occurs before pregnancy in 27% of women, during pregnancy for 33% of women, and in the postpartum phase for 40% of women [2].

Impacted mothers experience various adverse outcomes, including lower productivity and a higher risk of comorbidities like hypertension, diabetes mellitus, hyperlipidemia, and stroke [3,4]. Suicide accounts for approximately 20% of postpartum deaths, making it a leading cause of maternal mortality [5]. Effects on childhood growth outcomes include delayed cognitive development, lower social-emotional development, and higher chances of being underweight [6,7]. Affected families have higher medical and pharmaceutical spending during the first year post partum [8]. Left untreated, mother-child dyads incur significant health care expenditure, with estimates as high as US $32,000 per affected but untreated mother-child pair [9].

Despite the American College of Obstetricians and Gynecologists recommendations, screening and referral rates for postpartum depression (PPD) remain low [2,10]. In Medicaid populations, rates for screening and follow-up were less than 16% during pregnancy and 17% post partum. Commercially insured populations reported only 9% of pregnant women and 11% of postpartum women being screened [11]. Among those who screen positive, only 22% receive help [12].

Current evidence-based treatment approaches include both pharmacologic and nonpharmacologic interventions. Selective serotonin reuptake inhibitors (SSRIs) are commonly prescribed for PPD treatment. Systematic reviews of randomized controlled trials (RCTs) demonstrate the efficacy of SSRIs compared with placebo in women with PPD, although they fail to demonstrate superiority over other treatment modalities. Moreover, the evidence supporting SSRIs as a treatment modality for PPD is limited by the small number of RCTs, low representativeness of trial participants, underpowered samples, lack of long-term follow-up, and high dropout rates [13,14].

In 2019, the US Food and Drug Administration (FDA) approved the first treatment specifically for PPD. Brexanolone consists of a 60-hour continuous, in-hospital intravenous infusion. Significant practical and logistical barriers are associated with brexanolone use, including insurance coverage, childcare, excessive sedation, and loss of consciousness. Brexanolone is only indicated for moderate-to-severe PPD [15,16].

In 2023, the FDA approved zuranolone based on the results of 2 RCTs. The primary endpoint of both studies was the improvement in depressive symptoms using the total score from the 17-item Hamilton Depression Rating Scale (HAMD-17). In both studies, patients in the zuranolone arms showed significantly more improvement in their symptoms compared with those in the placebo arms (an improvement from baseline in HAMD-17 score of 17.8 vs 13.6 and 15.6 vs. 11.6). Several adverse reactions have been reported, including increased suicidal thoughts and behavior, somnolence and confusion, and impaired ability to drive. There is currently no data on the zuranolone’s effect on breastfed infants and limited data on milk production [1].

Nonpharmacologic interventions for PPD primarily focus on behavioral therapy. Meta-analyses demonstrate that Cognitive Behavioral Therapy (CBT) and Interpersonal Therapy (IPT) significantly improve short-term and long-term depressive symptomology compared with control, as measured by the Edinburgh Postnatal Depression Scale (EPDS) [17,18]. There is a wide consensus, including in discussions with regulators, that a 4 or more-point improvement in EPDS score is considered clinically meaningful [19,20]. There is evidence that other modalities, including Behavioral Activation Therapy (BAT) and Dialectical Behavioral Therapy (DBT), are also effective in this population [21,22]. However, the efficacy of psychological interventions is limited by the availability of therapists, insurance coverage, childcare, stigma, and transportation costs.

Evidence suggests that internet-enabled and mobile app–based psychological interventions yield improvements in depressive symptoms, although the benefit is contingent on adherence, which may be low if no human support is given [22-24]. Internet-based interventions hold several advantages over traditional, “face-to-face” therapy and telehealth, including enhanced flexibility, accessibility, convenience, lower cost, anonymity, and less stigma [25,26]. Due to these advantages, internet-based interventions hold promise for the postpartum population.

### Objective

This study evaluated the clinical efficacy of the self-guided MamaLift Plus digital therapeutic for the improvement of PPD symptoms in women who had recently given birth and had PPD, as assessed by the EPDS. MamaLift Plus is intended to be used as an adjunct to clinician‐managed outpatient care.

## Methods

### Trial Design

The SuMMER study was a double-blind, 8-week, randomized, placebo-controlled pivotal trial. The study was decentralized and conducted in the United States. Participants were randomized (2:1) to the MamaLift Plus intervention arm or sham control arm. This study was reported in line with the CONSORT (Consolidated Standards of Reporting Trials) guidelines [27].

### Participants

The study aimed to recruit participants who had recently given birth and had mild-to-moderate PPD. Participants were recruited remotely via social media ad campaigns on Meta platforms. Ads described the study as a “paid research study.” Ads were targeted to reach females within the target age range with interests in motherhood, parenting, or childcare Sex data was self-reported in the prescreener. Participants were asked whether they had given birth within the 3 months before the start of the study (live birth). Response options were “yes” and “no.”

### Eligibility Criteria

Key inclusion criteria are listed in Textbox 1.

**Defined criteria for inclusion in the SuMMER Study.**

- Participants must be 18 to 50 years of age at the time of enrollment.
- Participants must have given live birth within 3 months of enrollment.
- Participants must score greater than or equal to 13 but not exceeding 19 on the Edinburgh Postnatal Depression Scale.
- Depression diagnosis needs to be confirmed by a licensed behavioral health therapist or medical professional.
- Participants must answer “0/Never” or “1/Hardly Ever” to the self-harm question on Edinburgh Postnatal Depression Scale (Question #10).
- Participants must be willing to use a mobile app and own an iOS or android enabled mobile phone or device.
- Participants must have wireless internet connectivity in the home (or access to internet connectivity) and be willing to connect devices via a Wi-Fi network.
- Participants who had been previously diagnosed with serious mental illness or previously participated in a study conducted by Curio were excluded from the SuMMER study.

Participants were provided with a digital e-consent form (Multimedia Appendix 1) and scheduled to meet with a licensed mental health provider. The provider administered a diagnostic clinical interview based on the *DSM-5* (*Diagnostic and Statistical Manual of Mental Disorders, Fifth Edition*) criteria and a HAMD-17 to confirm a diagnosis of PPD. Participants who scored in the 8-23 range of the HAMD-17 were eligible to participate in the study [28].

The study coordinator who assigned participants to treatment arms did not have further involvement with the participants. The study monitor who enrolled participants also identified and provided outreach to participants who were nonconcordant. The study monitor did so in a masked fashion. The guidance given to participants did not differ based on the treatment arm, and no data could be edited.

### Interventions

MamaLift Plus is an 8-week digital therapeutic for the treatment of symptoms of PPD. MamaLift Plus can be used on a mobile device, such as a smartphone or tablet. MamaLift Plus delivers digital CBT, BAT, IPT, and DBT for PPD. As with traditional CBT delivered in a face-to-face format with a licensed mental health professional, MamaLift Plus uses personalized cognitive restructuring as the main therapeutic component to improve symptoms of PPD. The behavioral therapy content of MamaLift Plus is delivered via text, illustrations, video vignettes, and engaging exercises in 8 self-guided treatment modules. In addition, MamaLift Plus included features such as daily sleep, mood, and activity trackers. The main therapeutic component of MamaLift Plus is cognitive restructuring. MamaLift Plus demonstrated acceptability and usability in a previous human factors trial [29]. MamaLift Plus was developed by Curio Digital Therapeutics.

The sham control mimicked the features, functionality, and user experience of the treatment. It was designed to appear and feel similar to the treatment, but without the therapeutic or active ingredients that would induce the intended physiological or psychological effects. Specifically, the most important difference between participants in the 2 arms was that participants in the sham control app did not receive any CBT content. Sham control content paralleled the treatment arm with regard to the frequency of engaging with the app and the relative “workload” in each arm was similar. Participants were recommended to use their app daily, and they were informed that daily use of the application would require approximately 8 to 12 minutes per day for 8 weeks. Participants in both arms received automated “nudges” to remind them to use the SuMMER study app daily. Content for both apps was “frozen” during the trial.

Participants were required to complete assessments at protocol-specified intervals. Participants completed a baseline demographics questionnaire, health care usage questionnaire, mental health treatment received questionnaire, and prescribed medication use questionnaire on their first day in the app. Participants completed a health care usage questionnaire in the app every 2 weeks and an EPDS assessment every 4 weeks. Participants completed an end-of-treatment (EOT) EPDS, health care usage questionnaire, mental health treatment received questionnaire, and prescribed medication use questionnaire at EOT. Participants who completed all study-related activities received compensation.

### Outcomes

- The primary outcome was the proportion of participants whose EPDS improved by ≥4 points at their EOT assessment.
- The secondary endpoint was the proportion of participants whose EPDS score improved to <13 at their EOT assessment.
- A second secondary endpoint was the proportion of participants whose HAMD-17 scores improved at EOT.

The choice of primary endpoint is based on the consensus, including in discussions with regulators, that a 4 or more-point improvement in EPDS score is considered clinically meaningful [19,20]. The primary and secondary endpoints were self-assessed in the mobile app. The second secondary endpoint was assessed by a licensed mental health provider. The supportive EOT HAMD-17 was intended to be collected from all participants. However, most participants exited the study before EOT HAMD-17s could be initiated. Use was measured according to the completion of treatment modules and postbaseline assessments. Adverse event assessment was conducted by monitoring reserve keywords in the app and by an increase in EPDS score of more than 4 points. Reserve keywords are words or phrases that may indicate a declining mental state. AEs were monitored in the treatment and the sham control arm via a journal function using a free text option. No serious adverse events were reported during the study.

### Sample Size

Sample size calculations were based on a 2-sided α of .05 and a planned power of 80%. The endpoint is responder status as defined as an improvement in EPDS from baseline.

Previous trials suggest that the control response rate is 40% [30,31]. Study investigators hypothesized the response rate in the MamaLift Plus intervention arm would be 60%. The normal approximation to the binomial distribution was used to determine the required sample size. To detect a 20% delta (Þ), 210 participants would need to be enrolled. The study planned to randomize participants 2:1 into intervention (n=140) and control (n=70).

Since the study enrolled ~140 subjects, a post-hoc power calculation showed that a 25% delta can be detected with 80% power.

### Analysis Populations

The intention-to-treat (ITT) population included all randomized participants who started at least one module.

The full analysis set (FAS) is a subset of the ITT and included all participants randomized in the study who started at least 1 module and provided a postbaseline (either week 4 or end of treatment) EPDS assessment. All efficacy analyses were performed using Python 3.7 (Python Software Foundation) and Pandas 1.3.5 (Community).

### Randomization

Participants were randomized in a 2:1 ratio to the active (MamaLift Plus) and sham control arms. The randomization was stratified based on whether the participant had delivered her first child (primiparous) or whether she had previous live birth or births. Randomization was outsourced to a contract research organization. The authors specified that participants were to be randomized in a 2:1 ratio to the MamaLift Plus or sham control arm. The randomization was to be stratified based on “new mother” status (ie, whether the participant was primiparous or multiparous). The randomization code was developed and maintained by the contract research organization. Codes were stored in a secure environment. A study monitor provided enrollment support and outreach to nonconcordant participants. The study monitor was able to do so in a masked fashion. The guidance given to participants did not differ based on the treatment arm, and no data could be edited. Participants were also masked to arm assignment, as both applications had identical user interfaces, color schemes, and feature sets. Both applications were referred to as the “SuMMER study app” to protect participant masking. To further protect participant masking, the sham app included content of general interest to women in the postpartum period. Those assessing outcomes and analyzing the data were also masked to arm assignment.

### Statistical Methods

All participants with missing postbaseline primary endpoint assessments were treated as nonresponders in the primary efficacy analysis.

The primary comparison was a *z* test of proportions between the MamaLift Plus and sham control arm using the normal approximation to the binomial distribution. The *z* test for proportions evaluates whether observed differences between 2 groups represent a real difference in the populations they were drawn from, or if the difference could reasonably be attributed to random sampling variation. The test statistic, a *z* score, is calculated using the formula: *z*=(p1-p2)/sqrt (p(1-p) (1/n1+1/n2)), where p is the pooled sample proportion.

For assessing mean improvement from baseline, a *t* test was used.

In this study, responder rates were compared between participants in the MamaLift Plus arm and the sham control arm. The proportion in each arm was the percent of responders where a responder is defined as a subject whose baseline EPDS score improves by ≥4 points by EOT. This analysis was performed in the ITT and FAS populations. This analysis was repeated for a subset of women who became mothers for the first time, that is, gave birth to their first child. The primary endpoint was tested first at α=.05 (2-sided). For all other analyses (secondary endpoint and other analysis sets), no adjustments for multiplicity were made. Hence, *P* values are considered nominal.

### Ethical Considerations

This study protocol received human subject ethics review and approval from the Brany institutional review board before data collection (ethics board study ID number CU-T-003). All participants provided informed consent electronically. The informed consent included information about the purpose, voluntary participation, withdrawal conditions, length of participation, key study procedures, payment terms, risks and benefits, alternatives to study participation, costs, and confidentiality. The informed consent also included contact information for study personnel and information about free federal mental health resources, including national mental health hotlines. The informed consent included a statement describing that participants had the ability to opt out of participation in the study at any point at no penalty to themselves. Data was deidentified during analysis to safeguard participant information and reduce the risk of bias. Participants received a US $100 digital Amazon gift card upon completing all study activities.

## Results

From April 18 to May 24, 2023, a total of 2177 participants were assessed for eligibility. Out of these, 2035 were determined to be ineligible, 637 of whom were ineligible because they completed the study screener after the last participant in, and 1398 did not meet the inclusion criteria. A total of 142 participants were enrolled. Following assessment with the licensed mental health provider, 1 participant was recalled from the study, and 141 participants were randomized. Out of 141 participants, 11 (5 from the intervention group and 6 from the digital placebo group) did not provide the primary endpoint assessment, resulting in 130 participants being included in the FAS analysis. Finally, 95 participants were included in the final ITT analysis of the intervention arm, and 46 were included in the ITT analysis for the control arm.

**Figure 1:**
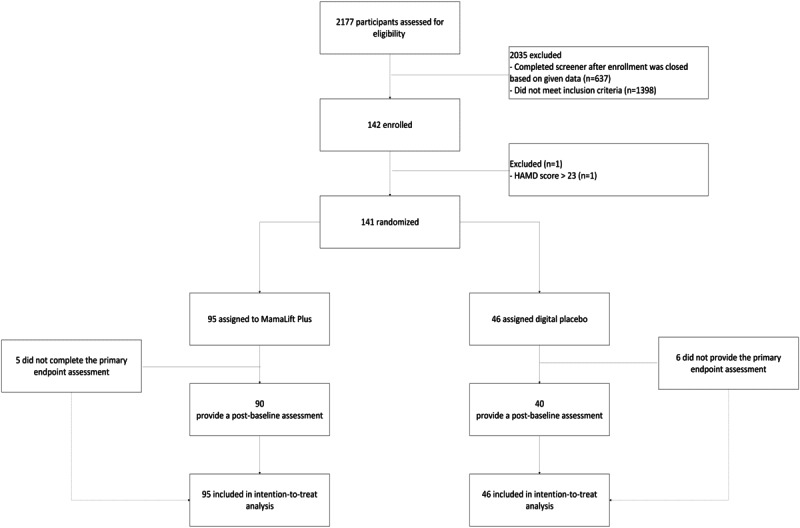
CONSORT (Consolidated Standards of Reporting Trials) flow diagram. HAMD: Hamilton Rating Scale for Depression.

Results from the baseline demographics questionnaire are given below in Table 1. Participants in both arms had similar mean ages and comparable marital statuses. Participants with a bachelor’s degree were the most represented overall, with 53% (50/95) in the intervention arm and 48% (22/46) in the control arm. Parity was not significantly different in either arm. Only 136 participants provided ethnicity information in their baseline demographics questionnaire (see the CONSORT flow diagram in Figure 1) [27].

Moreover, the participants of this study were representative of the United States in terms of state-wise distribution, with the highest representation being from populous states like Texas, California, New York, Florida, and New Jersey. A complete table of state-wise distribution within each treatment arm is available in Multimedia Appendix 1.

The primary efficacy endpoint was the proportion of participants whose EPDS scores improved by ≥4 points at the EOT assessment. The secondary efficacy endpoint was the proportion of participants whose EPDS scores improved to <13. Key subgroups were defined by “New Mom” status and “Anti-depressive use” status. Responder analysis was performed for both ITT and FAS populations. The ITT population included all randomized participants who started at least one module. The FAS included all participants randomized in the study who started at least 1 module and provided a postbaseline (either week 4 or [EOT]) EPDS assessment.

**Table 1:** Baseline demographics of the intent-to-treat population.

Characteristics		MamaLift plus (n=95)	Sham control (n=46)
Age (years), mean (SD)		32.39 (5.54)	30.74 (5.54)
**Marital status, n (%)**			
	Divorced	1 (1.05)	0 (0)
	I prefer not to answer	1 (1.05)	0 (0)
	Married or domestic partnership	73 (76.8)	38 (82.6)
	Single, never married	16 (16.8)	6 (13.04)
	Other	4 (4.2)	2 (4.3)
**Education level, n (%)**			
	Bachelor’s degree	50 (52.6)	22 (47.8)
	Doctorate degree	2 (2.1)	1 (2.2)
	High school diploma or GED^a^	25 (26.3)	7 (15.2)
	I prefer not to answer	1 (1.05)	1 (2.2)
	Master’s degree	11 (11.6)	13 (28.3)
	Professional degree beyond bachelor’s degree	2 (2.1)	0 (0)
	Not applicable	4 (4.2)	2 (4.3)
**New mom, n (%)**			
	New mom (yes)	60 (63.1)	31 (67.3)
**Race, n (%)**			
	Asian	3 (3.3)	3 (6.8)
	Black or African American	39 (42.4)	18 (40.9)
	Hispanic (Latinx)	13 (14.1)	4 (9.1)
	White	34 (37)	17 (38.6)
	American Indian or Alaska Native	2 (2.2)	0 (0)
	I prefer not to answer	1 (1.1)	2 (4.6)

^a^GED: General educational development.

Analysis for the ITT population was performed on 141 participants, including 95 in the intervention arm and 46 in the control arm (Table 2). Among participants in the intervention arm, 82 out of 95 (86.3%) demonstrated an EPDS improvement of ≥4 points, compared with 11 out of 46 (23.9%) in the control arm (*P*<.0001). The findings in each subgroup stratum are consistent with the overall treatment arm results and are in favor of the MamaLift Plus treatment. Of the 95 participants in the MamaLift Plus arm, 60 identified as new moms and 35 did not identify as new moms. A total of 51 of the 60 (85%) new moms in the MamaLift Plus arm demonstrated an improvement of ≥4 points, compared with only 25.8% (8/31) of new moms in the sham control arm. Thus, efficacy results were similar for “New Mom=Yes” and “New Mom=No.”

**Table 2:** Efficacy analysis of responder results for the intent-to-treat population.

Population or subgroup		MamaLift plus (n=95)	Sham control (n=46)	*P* value (α=.05)
**Primary endpoint: improvement of 4+ points, n/n (%)**				
	All moms	82 (86.3)	11 (23.9)	<.0001
	New mom=yes	51/60 (85)	8/31 (25.8)	<.001
	New mom=no	31/35 (88.6)	3/15 (20)	<.001
	Antidepressive=yes	15/16 (93.8)	3/14 (21.4)	<.001
	Antidepressive=no	67/79 (84.8)	8/32 (25)	<.001
**Secondary endpoint: improvement to <13 EPDS^a^,** **n** **(%)**				
	All moms	79 (83.2)	15 (32.6)	<.0001

^a^EPDS: Edinburgh Postnatal Depression Scale.

Of the 95 participants in the MamaLift plus arm, 16 reported taking antidepressants and 79 reported not taking antidepressants. A total of 15 of the 16 (93.8%) participants in the MamaLift Plus arm who reported taking antidepressants demonstrated an improvement of ≥4 points, compared with only 21.4% (3/14) of the participants in the sham control arm. Of the 79 participants in the MamaLift Plus arm who reported not taking antidepressants, 67 (84.8%) demonstrated an improvement of ≥4 points, compared with only 25% in the sham control arm. Thus, efficacy results were similar in both subgroup analyses. This analysis demonstrates that the proportion of participants in the MamaLift Plus arm who achieved a clinically meaningful improvement of ≥4 points was comparable in those who were taking antidepressants and those who were not. Similarly, a much lower proportion of participants in the sham control arm achieved the same clinically meaningful improvement, regardless of antidepressant use status. Among participants in the MamaLift Plus arm, 79 out of 95 (83.2%) also met the secondary endpoint of demonstrating an improvement to <13 EPDS (*P*<.0001). This is compared with only 15 (32.6%) participants in the sham control arm who met the same endpoint (Table 2).

Responder analysis for the FAS population was performed on 130 participants (Table 3). Results show that, among intervention arm participants (n=90), 82 out of 90 (91.1%) demonstrated an EPDS improvement of ≥4 points, compared with only 11 out of 40 (27.5%) in the sham control arm (*P*<.0001). Among new moms in the MamaLift Plus arm (n=56), 51 out of 56 moms (91.1%) achieved an improvement of ≥4 points, compared with only 28.6% (8/28) of new moms in the sham control arm.

**Table 3:** Efficacy analysis of responder results for the full analysis set population.

Population or subgroup			MamaLift plus (n=90)		Sham control (n=40)		*P* value (α=.05)
**Primary endpoint: improvement of 4+ points, n (%)**							
	All moms	82/90 (91.1)		11/40 (27.5)		<.0001	
	New mom=yes	51/56 (91.1)		8/28 (28.6)		<.001	
	New mom= no	31/34 (91.2)		3/12 (25)		<.001	
**Secondary endpoint: improvement to <13 EPDS^a^, n/n (%)**							
	All moms	79/90 (87.7)		15/40 (37.5)		<.0001	

^a^EPDS: Edinburgh Postnatal Depression Scale.

A total of 79 of the 90 participants in the MamaLift Plus arm (87.7%) also met the secondary endpoint of demonstrating an improvement to <13 EPDS. Comparatively, only 15 of the 40 participants in the sham control arm demonstrated an improvement to <13 on the EPDS.

A *t* test for mean improvement from baseline was performed for both ITT and FAS populations. In the ITT population, the mean improvement from baseline for participants in the MamaLift Plus arm was 8.34 (SD 4.84) points. This is substantially less than the mean 1.89 (SD 3.22) point improvement achieved in the sham control arm (*P*<.0001). In the FAS population, the mean improvement from baseline for participants in the MamaLift Plus arm was 8.8 (4.54) points. Comparatively, the mean improvement from baseline for participants in the sham control arm was only 2.18 (SD 3.37) points (*P*<.0001).

Overall, 43 participants completed the EOT HAMD-17, with 33 in the MamaLift Plus arm and 10 in the sham control arm. Of those who completed the EOT HAMD-17, the mean improvement from baseline for participants in the MamaLift Plus arm was 12.1 (SD 5.87) points, compared with 9.4 (SD 9.52) points in the sham control arm (*P*=.28). Although the sample is limited, a trend can be discerned in which participants in the MamaLift Plus arm achieve a greater improvement in HAMD-17 EOT scores compared with participants in the sham control arm.

A total of 4 AEs were identified, triaged, and documented in the study: 2 were in the MamaLift Plus arm and 2 were in the sham control arm. Of the 4 AEs, 2 were identified via the use of reserve keywords. Both were in the MamaLift Plus arm. As per the study protocol, participant use of specific keywords in the application triggered an alert to the study staff. The remaining 2 AEs were identified based on EPDS scores increasing by 4 or more points from baseline. Both were in the sham control arm. After a joint evaluation by the investigator and participants’ clinicians, these participants continued the study.

## Discussion

### Overview

This study evaluated MamaLift Plus’s efficacy in reducing symptoms of depression in women diagnosed with PPD. This double-blind, randomized, placebo-controlled pivotal trial examined the impact of MamaLift Plus versus sham control on symptoms of depression, as measured by the EPDS.

### Principal Findings

Responder analysis showed statistically significant and clinically meaningful differences between MamaLift Plus participants and sham control arm participants. In the analysis of the ITT population, 86.3% (82/95) of MamaLift Plus arm participants achieved an improvement of ≥4 points, compared with only 23.9% (11/46) in the sham control arm. Results were consistent among women who were new moms and those who were not. A total of 85% (51/60) of new moms and 88.6% (31/35) of women who were not new moms achieved an improvement of ≥4 points in the MamaLift Plus arm. This suggests that the combination of CBT, IPT, DBT, and BAT in a digital intervention is effective for reducing depressive symptoms associated with PPD. In addition, a greater proportion of intervention arm participants in the FAS analysis demonstrated an improvement of 4+ points compared with control arm participants. HAMD-17 results were encouraging and displayed a similar dose-response effect with participants in the intervention arm displaying improvement in depressive symptoms as early as 4 weeks.

### Comparison to Previous Work

Until 2019, there were no FDA-approved treatments specifically for PPD. The advent of brexanolone was a significant step forward, although several practical barriers remain for patients interested in taking the 60-hour continuously intravenously administered drug. In 2023, the FDA approved zuranolone. Zuranolone is taken orally daily for 14 days. However, zuranolone also has significant risks, including lack of efficacy data beyond 42 days, sedation, and potential suicidal thoughts. Moreover, both of these treatments are indicated for moderate-to-severe PPD [15,16], leaving a significant gap for women with mild-to-moderate PPD. Systematic reviews suggest that behavioral therapies, including CBT, IPT, DBT, and BAT are safe and efficacious in reducing symptoms of mild-to-moderate PPD [17,18,21,22]. Other digital interventions have demonstrated promising usability and are being evaluated in RCTs, including German studies of the apps SmartMoms and Smart-e-Moms [32,33]. However, these apps are primarily focused on promoting information about PPD, screening for PPD, or providing therapist-guided support. Importantly, they were not intended for women with a diagnosis of PPD. Other tools from New Zealand and Australia have demonstrated an impact on stress, but not symptoms of depression [34]. However, patients still face significant barriers to engaging with these therapies, including stigma, lack of time, and a well-documented shortage of qualified mental health providers in the United States to deliver these therapies.

This study examined the efficacy of a self-guided, mobile app-delivered, combined CBT, IPT, BAT, and DBT program in the postpartum population. This study provided evidence that MamaLift Plus is more efficacious than a sham control in improving symptoms of depression in women diagnosed with PPD, as measured by the EPDS. Our findings corroborate earlier studies showing behavioral therapy modalities like CBT, IPT, BAT, and DBT are efficacious in improving symptoms of depression. Moreover, the findings of this pivotal trial demonstrate that the benefits of these behavioral therapy modalities are translated to a digital medium with high fidelity and promising results.

### Strengths and Limitations

First, the main limitation of this study is that it does not provide insight into the long-term effect after the 8-week intervention is over. Second, by the nature of the trial being decentralized, we may anticipate patients receiving MamaLift Plus in a clinical setting behaving differently than the ones in the SuMMER study. Attempts were made to mitigate the impact of this limitation by informing participants that they should continue engaging with any therapies or treatments that are recommended by their physician. Third, even though the study met its primary endpoint, the small number of participants who completed a HAMD-17 is a limitation. This had a minimal influence on the results, although a greater number of HAMD-17 completers would have strengthened the study’s conclusions. Fourth, the FAS analysis only included 12 participants who were not new moms in the control arm. Fifth, there was no test for blindness, without which the study investigators cannot be certain that control arm participants did not alter their behavior due to knowledge of their allocation. Efforts were made to mitigate this limitation. As described earlier, the sham control mimicked the features, functionality, and user experience of the treatment. The sham control was designed to appear and feel like the treatment, but without the therapeutic ingredients that would induce the intended psychological effects. No inferences can be made other than the ones reported in this study.

The representativeness of the study sample is a significant strength of this study and suggests that the findings may be generalizable. The average age and marital statuses were comparable. A greater proportion of control arm participants held master’s degrees compared to the intervention arm. Given the lower educational attainment in the intervention arm and the correlation between educational attainment and access to mental health resources, it was more impressive that the intervention arm displayed greater reductions in depressive symptoms. The racial composition of both arms was comparable. Moreover, states with the highest representation in the study were also the most populous states. Comparable proportions of intervention arm and control arm participants reported taking prescription medications at baseline and EOT.

### Future Directions

Future studies will include 30- or 90-day follow-up periods to determine long-term effects.

### Conclusions

The results of this pivotal trial demonstrate that MamaLift Plus is efficacious in reducing symptoms of depression associated with PPD. MamaLift Plus may be an important treatment option for women with symptoms of PPD. Future studies may evaluate the effectiveness of MamaLift Plus in higher-risk participants or participants receiving care at an obstetrics and gynecology practice.

## Appendix

Multimedia Appendix 1 State-wise Distribution of Participants.

Multimedia Appendix 2 CONSORT eHEALTH checklist (V 1.6.2).

## Abbreviations

BAT: Behavioral Activation Therapy
CBT: Cognitive Behavioral Therapy
CONSORT: Consolidated Standards of Reporting Trials
DBT: Dialectical Behavior Therapy
DSM-5: Diagnostic and Statistical Manual of Mental Disorders, Fifth Edition
EOT: end-of-treatment
EPDS: Edinburgh Postnatal Depression Scale
FAS: full analysis set
FDA: Food and Drug Administration
HAMD-17: Hamilton Rating Scale for Depression
IPT: Interpersonal Therapy
ITT: intent-to-treat
PPD: postpartum depression
RCT: randomized controlled trial
SSRI: selective serotonin reuptake inhibitors

## Data Availability

The datasets generated or analyzed during this study are available from the corresponding author on reasonable request.
