# Supplementary material for: Evaluating the Efficacy of MamaLift Plus Digital Therapeutic Mobile App for Postpartum Depression (SuMMER): Randomized, Placebo-Controlled Pivotal Trial
Source: J Med Internet Res. 2025 Jul 1;27:e69050. doi: 10.2196/69050 (PMC12264441; doi:10.2196/69050)
Supplement: Multimedia Appendix 1 [file jmir_v27i1e69050_app1.docx]

State-wise Distribution of Participants

| ***State*** | ***Sham Control*** | ***MamaLift Plus*** |
| --- | --- | --- |
| ***Indiana*** | *2 (4.3%)* | *2 (2.1%)* |
| ***Kentucky*** | *1 (2.2%)* | *0 (0%)* |
| ***Louisiana*** | *0 (0%)* | *2 (2.1%)* |
| ***Michigan*** | *1 (2.2%)* | *4 (4.2%)* |
| ***Texas*** | *3 (6.5%)* | *12 (12.6%)* |
| ***Alabama*** | *0 (0%)* | *1 (1.1%)* |
| ***Arizona*** | *1 (2.2%)* | *2 (2.1%)* |
| ***California*** | *8 (17.4%)* | *12 (12.6%)* |
| ***Colorado*** | *0 (0%)* | *2 (2.1%)* |
| ***Florida*** | *2 (4.3%)* | *7 (7.4%)* |
| ***Georgia*** | *1 (2.2%)* | *0 (0%)* |
| ***Idaho*** | *0 (0%)* | *1 (1.1%)* |
| ***Illinois*** | *0 (0%)* | *4 (4.2%)* |
| ***Iowa*** | *1 (2.2%)* | *0 (0%)* |
| ***Maryland*** | *4 (8.7%)* | *2 (2.1%)* |
| ***Massachusetts*** | *0 (0%)* | *2 (2.1%)* |
| ***Mississippi*** | *1 (2.2%)* | *0 (0%)* |
| ***Nevada*** | *1 (2.2%)* | *2 (2.1%)* |
| ***New Jersey*** | *3 (6.5%)* | *4 (4.2%)* |
| ***New York*** | *6 (13%)* | *10 (10.5%)* |
| ***North Carolina*** | *0 (0%)* | *1 (1.1%)* |
| ***North Dakota*** | *0 (0%)* | *1 (1.1%)* |
| ***Ohio*** | *2 (4.3%)* | *2 (2.1%)* |
| ***Oklahoma*** | *0 (0%)* | *1 (1.1%)* |
| ***Oregon*** | *0 (0%)* | *2 (2.1%)* |
| ***Pennsylvania*** | *1 (2.2%)* | *5 (5.3%)* |
| ***South Carolina*** | *1 (2.2%)* | *3 (3.2%)* |
| ***Tennessee*** | *0 (0%)* | *1 (1.1%)* |
| ***Utah*** | *1 (2.2%)* | *1 (1.1%)* |
| ***Virginia*** | *0 (0%)* | *3 (3.2%)* |
| ***Washington*** | *1 (2.2%)* | *0 (0%)* |
| ***Wisconsin*** | *1 (2.2%)* | *0 (0%)* |
| ***Wyoming*** | *1 (2.2%)* | *0 (0%)* |
