# Supplementary material for: Evaluating the Efficacy of MamaLift Plus Digital Therapeutic Mobile App for Postpartum Depression (SuMMER): Randomized, Placebo-Controlled Pivotal Trial
Source: J Med Internet Res. 2025 Jul 1;27:e69050. doi: 10.2196/69050 (PMC12264441; doi:10.2196/69050)
Supplement: Multimedia Appendix 2 [file jmir_v27i1e69050_app2.pdf]

CONSORT-EHEALTH Checklist V1.6.2 Report	Manuscript Number	69050
**(based on CONSORT-EHEALTH V1.6), available at [http://tinyurl.com/consort-ehealth-v1-6].**		
**Date completed**		
12/9/2024 21:24:45		
**by**		
Indira Malladi		
Efficacy of MamaLift Plus Digital Therapeutic Mobile Application in Patients with Postpartum Depression (SuMMER): A Nationwide, Randomized, Placebo-Controlled Pivotal Trial		
TITLE		
**1a-i) Identify the mode of delivery in the title**		
"Efficacy of MamaLift Plus Digital Therapeutic Mobile Application in Patients with Postpartum Depression (SuMMER): A Nationwide, Randomized, Placebo-Controlled Pivotal Trial."		
**1a-ii) Non-web-based components or important co-interventions in title**		
No. It is not relevant because there are no co-interventions.		
**1a-iii) Primary condition or target group in the title**		
"Patients with Postpartum Depression."		
ABSTRACT		
**1b-i) Key features/functionalities/components of the intervention and comparator in the METHODS section of the ABSTRACT**		
"MamaLift Plus is a self-guided 8-week digital therapeutic for symptomatic treatment for PPD. MamaLift Plus can be used on a mobile device, such as a smartphone or tablet. MamaLift Plus delivers digital Cognitive Behavioral Therapy (CBT), Behavioral Activation Therapy (BAT), Interpersonal Therapy (IPT), and Dialectical Behavior Therapy (DBT) for PPD."		
**1b-ii) Level of human involvement in the METHODS section of the ABSTRACT**		
"MamaLift Plus is a self-guided 8-week digital therapeutic."		
**1b-iii) Open vs. closed, web-based (self-assessment) vs. face-to-face assessments in the METHODS section of the ABSTRACT**		
"Participants were recruited online between April 18 and May 24, 2023. Eligible participants attended a virtual, face-to-face assessment with a licensed mental health provider to confirm a diagnosis of PPD." "Primary and secondary endpoints were self-assessed."		
**1b-iv) RESULTS section in abstract must contain use data**		
Patient use/engagement with the app was measured according to completion of treatment modules and post-baseline assessments. "Analysis for the Intent-to-Treat (ITT) population included 141 participants." "Analysis for the Full Analysis Set (FAS) included 130 participants."		
**1b-v) CONCLUSIONS/DISCUSSION in abstract for negative trials**		
This was not a negative trial. Primary outcomes are discussed in the results and conclusions sections of the abstract.		
INTRODUCTION		
**2a-i) Problem and the type of system/solution**		
The goals and intended patient population are described with the following: "This study evaluated the clinical efficacy of the MamaLift Plus digital therapeutic for the improvement of PPD symptoms in women who had recently given birth and had PPD, as assessed by the Edinburgh Postnatal Depression Scale (EPDS)." The incorporation of MamaLift Plus into a healthcare program is described with: "MamaLift Plus is intended to be used as an adjunct to clinician-managed outpatient care."		
**2a-ii) Scientific background, rationale: What is known about the (type of) system**		
Current knowledge of type of system is described with, "Non-pharmacologic interventions for PPD primarily focus on behavioral therapy. Meta-analyses demonstrate that Cognitive Behavioral Therapy (CBT) and Interpersonal Therapy (IPT) significantly improve short-term and long-term depressive symptomology compared to control." The justification for this study, stakeholder population, and potential impact are described in the following statement: "Evidence suggests that internet-enabled and mobile application-based psychological interventions yield improvements in depressive symptoms." "Internet-based interventions hold several advantages over traditional, “face-to-face” therapy and telehealth, including enhanced flexibility, accessibility, convenience, lower cost, anonymity, and less stigma." "Internet-based interventions hold promise for the postpartum population." The choice of comparator is described in detail in the Methods section.		
**Does your paper address CONSORT subitem 2b?**		
Yes. IRB approval is discussed in the Methods section instead of the Introduction section.		
METHODS		
**3a) CONSORT: Description of trial design (such as parallel, factorial) including allocation ratio**		
Yes. "Participants were randomized (2:1 ratio with block size of 9) to the MamaLift Plus intervention arm or sham control arm."		
**3b) CONSORT: Important changes to methods after trial commencement (such as eligibility criteria), with reasons**		
There were no changes after trial commencement that impacted study design.		
**3b-i) Bug fixes, Downtimes, Content Changes**		
No, there were no significant bug fixes, downtimes, or content changes.		
**4a) CONSORT: Eligibility criteria for participants**		
Yes. Eligibility criteria and informed consent procedures are described in the manuscript. Recruitment methods are described with: "Participants were recruited online via social media campaigns." Face-to-face components are described in: "The provider administered a Hamilton Rating Scale for Depression (HAMD) to confirm a diagnosis of PPD." The degree to which the study team got to know the participant is described in, "The study coordinator who assigned participants to treatment arms did not have further involvement with the participants. The study monitor who enrolled participants also identified and provided outreach to participants who were non-concordant. The study monitor did so in a masked fashion."		
**4a-i) Computer / Internet literacy**		
"Participants must be willing to use a mobile app and own an iOS or android enabled mobile phone or device. Participants must have wireless internet connectivity in the home (or access to internet connectivity) and be willing to connect devices via a Wi-Fi network<br>Participants who had been previously diagnosed with Serious Mental Illness (SMI) or previously participated in a study conducted by Curio were excluded from the SuMMER study."		
**4a-ii) Open vs. closed, web-based vs. face-to-face assessments:**		
Recruitment methods are given in, "Participants were recruited online via social media campaigns." Face-to-face components are described in, "The provider administered a Hamilton Rating Scale for Depression (HAMD) to confirm a diagnosis of PPD." The degree to which the study team got to know the participant is described in, "The study coordinator who assigned participants to treatment arms did not have further involvement with the participants. The study monitor who enrolled participants also identified and provided outreach to participants who were non-concordant. The study monitor did so in a masked fashion." Email confirmation and phone call were used to prevent participants with multiple identities from participating.		
**4a-iii) Information giving during recruitment**		
Informed consent procedures are described with the following text: "Participants were provided with a digital e-consent form." Information given during recruitment, specifically during the informed consent process, is given in the Outcomes section.		
**4b) CONSORT: Settings and locations where the data were collected**		
Yes. "The primary and secondary endpoints were self-assessed in the mobile application." Reporting how institutional affiliations are displayed is not relevant for this study because there were no institutional affiliations.		
**4b-i) Report if outcomes were (self-)assessed through online questionnaires**		
Yes. "The primary and secondary endpoints were self-assessed in the mobile application."		
**4b-ii) Report how institutional affiliations are displayed**		
No. Reporting how institutional affiliations are displayed is not relevant for this study because there were no institutional affiliations.		
**5) CONSORT: Describe the interventions for each group with sufficient details to allow replication, including how and when they were actually administered**		
**5-i) Mention names, credential, affiliations of the developers, sponsors, and owners**		
Yes. "Curio Digital Therapeutics developed MamaLift Plus."		
**5-ii) Describe the history/development process**		

The development of MamaLift Plus has been described in detail in previous publications, which are referenced in this manuscript with the following text, "MamaLift Plus demonstrated acceptability and usability in a previous human factors trial."		
**5-iii) Revisions and updating**		
Yes. "Content for both apps was “frozen” during the trial." We have not included the build number in the manuscript because it is not relevant.		
**5-iv) Quality assurance methods**		
No, the manuscript does not address this subitem. The development and formative evaluation of MamaLift Plus content has been described in previously published articles, and they are referenced in this manuscript.		
**5-v) Ensure replicability by publishing the source code, and/or providing screenshots/screen-capture video, and/or providing flowcharts of the algorithms used**		
No, the paper does not address subitem 5-v. In order to protect Curio's intellectual property, algorithms or source code associated with MamaLift Plus will not be published.		
**5-vi) Digital preservation**		
No, the paper does not address subitem 5-vi because MamaLift Plus is not currently commercially available in the app store/Google Play store. It will become available in 2025.		
**5-vii) Access**		
Yes. "MamaLift Plus can be used on a mobile device, such as a smartphone or tablet." The sham app can also be used on a mobile device. This is explained in the following statement: "The sham control mimicked the features, functionality, and user experience of the treatment." Participants were compensated for their participation. This is explained in the following statement: "Participants who completed all study-related activities received compensation."		
**5-viii) Mode of delivery, features/functionalities/components of the intervention and comparator, and the theoretical framework**		
Yes. "MamaLift Plus can be used on a mobile device, such as a smartphone or tablet. MamaLift Plus delivers digital CBT, BAT, IPT, and DBT for PPD. The behavioral therapy content of MamaLift Plus is delivered via text, illustrations, video vignettes, and interactive exercises. Additionally, MamaLift Plus included features such as daily sleep, mood, and activity trackers."<br><br>The sham control is described with: "The sham control mimicked the features, functionality, and user experience of the treatment. It was designed to appear and feel similar to the treatment, but without the therapeutic or active ingredients that would induce the intended physiological or psychological effects."		
**5-ix) Describe use parameters**		
Yes. "Participants were recommended to use their app daily, and they were informed that daily use of the application would require approximately eight to twelve minutes per day for eight weeks." The content in MamaLift Plus is structured as eight treatment modules intended to be completed over a period of eight to nine weeks.		
**5-x) Clarify the level of human involvement**		
There was no human involvement in the intervention or co-intervention. The self-guided nature of the MamaLift Plus intervention is described here: "The behavioral therapy content of MamaLift Plus is delivered via text, illustrations, video vignettes, and interactive exercises in eight self-guided treatment modules. "		
**5-xi) Report any prompts/reminders used**		
"Participants in both arms received automated “nudges” to remind them to use the SuMMER study app daily." SMS nudge language was the same for participants in both arms.		
**5-xii) Describe any co-interventions (incl. training/support)**		
No co-interventions were provided.		
**6a) CONSORT: Completely defined pre-specified primary and secondary outcome measures, including how and when they were assessed**		
Yes, we have completely defined pre-specified primary and secondary outcome measures, including how and when they were assessed. The relevant text in the manuscript is the following: "The primary outcome was the proportion of participants whose EPDS improved by ≥4 points at their EOT assessment. The secondary endpoint was the proportion of participants whose EPDS score improved to <13 at their EOT assessment."		
**6a-i) Online questionnaires: describe if they were validated for online use and apply CHERRIES items to describe how the questionnaires were designed/deployed**		
We did not include this because the Edinburgh Postnatal Depression Scale (EPDS) has been extensively validated in separate studies, including for online use.		
**6a-ii) Describe whether and how “use” (including intensity of use/dosage) was defined/measured/monitored**		
This question is more relevant for a pharmacological agent, whereas our study Hence, only parts of this question are applicable. Recommended use is approximately ten minutes per day, as determined by prior studies. Use/engagement with the app was measured according to completion of treatment modules and post-baseline assessments.		
**6a-iii) Describe whether, how, and when qualitative feedback from participants was obtained**		
Qualitative feedback was not obtained.		
**6b) CONSORT: Any changes to trial outcomes after the trial commenced, with reasons**		
Yes. "The primary and secondary endpoints were self-assessed in the mobile application." Reporting how institutional affiliations are displayed is not relevant for this study because there were no institutional affiliations.		
**7a) CONSORT: How sample size was determined**		
**7a-i) Describe whether and how expected attrition was taken into account when calculating the sample size**		
The endpoint is binary, and the comparison is between two proportions. The estimated delta was obtained from prior studies, which already incorporated attrition. Thus, the delta used for our calculations had already accounted for attrition, and consequently, no further adjustments were required.		
**7b) CONSORT: When applicable, explanation of any interim analyses and stopping guidelines**		
Yes, we have completely defined pre-specified primary and secondary outcome measures, including how and when they were assessed. The relevant text in the manuscript is the following: "The primary outcome was the proportion of participants whose EPDS improved by ≥4 points at their EOT assessment. The secondary endpoint was the proportion of participants whose EPDS score improved to <13 at their EOT assessment."		
**8a) CONSORT: Method used to generate the random allocation sequence**		
"Participants were randomized (2:1 ratio with block size of 9) to the MamaLift Plus intervention arm or sham control arm. A randomization table was used to allocate participants to comparison arms."		
**8b) CONSORT: Type of randomisation; details of any restriction (such as blocking and block size)**		
"2:1 ratio with block size of 9"		
**9) CONSORT: Mechanism used to implement the random allocation sequence (such as sequentially numbered containers), describing any steps taken to conceal the sequence until interventions were assigned**		
"A randomization table was used to allocate participants to comparison arms."		
**10) CONSORT: Who generated the random allocation sequence, who enrolled participants, and who assigned participants to interventions**		
An independent group (HITLab) was responsible for the random code and their study coordinator assigned the digital app or digital placebo to participants.		
**11a) CONSORT: Blinding - If done, who was blinded after assignment to interventions (for example, participants, care providers, those assessing outcomes) and how**		
**11a-i) Specify who was blinded, and who wasn’t**		
Participants, study monitors, and those assessing outcomes were blinded to assigned intervention. "A study monitor provided enrollment support and outreach to non-concordant participants. The study monitor was able to do so in a masked fashion." Participants were also masked to arm assignment, as both applications had identical user interfaces, color schemes, and feature sets. Both applications were referred to as the “SuMMER study app." Those assessing outcomes and analyzing the data were also masked to arm assignment."		
**11a-ii) Discuss e.g., whether participants knew which intervention was the “intervention of interest” and which one was the “comparator”**		
"Both applications had identical user interfaces, color schemes, and feature sets. Both applications were referred to as the “SuMMER study app” to protect participant masking. To further protect participant masking, the sham app included content of general interest to women in the postpartum period."		
**11b) CONSORT: If relevant, description of the similarity of interventions**		
"The sham control mimicked the features, functionality, and user experience of the treatment. It was designed to appear and feel similar to the treatment, but without the therapeutic or active ingredients that would induce the intended physiological or psychological effects. Specifically, the most important difference between participants in the two arms was that participants in sham control app did not receive any CBT content. Sham control content paralleled the treatment arm with regards to frequency of engaging with the app and the relative “workload” in each arm was similar."		
**12a) CONSORT: Statistical methods used to compare groups for primary and secondary outcomes**		
"The endpoint is responder status as defined as an improvement in EPDS from baseline."		

**12a-i) Imputation techniques to deal with attrition / missing values**		
"All participants with missing post-baseline primary endpoint assessments were treated as non-responders in the primary efficacy analysis."		
**12b) CONSORT: Methods for additional analyses, such as subgroup analyses and adjusted analyses**		
"Key subgroups were defined by “New Mom” status and “Anti-depressive use” status."		
RESULTS		
**13a) CONSORT: For each group, the numbers of participants who were randomly assigned, received intended treatment, and were analysed for the primary outcome**		
Yes. The number of participants who were randomized, received intended treatment, and were analyzed is given in the manuscript. The relevant text is the following: "Analysis for the ITT population was performed on 141 participants, including 95 in the intervention arm and 46 in the control arm (Table 3)." "Responder analysis for the FAS population was performed on 130 participants (Table 4)."		
**13b) CONSORT: For each group, losses and exclusions after randomisation, together with reasons**		
Yes. A CONSORT Flow diagram is included in Figure 1. "141 participants were randomized. 11 participants (5 from the intervention group and 6 from the digital placebo group) did not provide the primary endpoint assessment, resulting in 130 participants being included in the FAS analysis. 95 participants are included in the final ITT analysis of the intervention arm. 46 are included in the ITT analysis for the control arm."		
**13b-i) Attrition diagram**		
This subitem is not relevant to our study because participants were expected to discontinue app use after completing eight treatment modules.		
**14a) CONSORT: Dates defining the periods of recruitment and follow-up**		
There were no critical secular events during the recruitment or study period. The trial did not end early.		
**14a-i) Indicate if critical “secular events” fell into the study period**		
There were no critical secular events during the recruitment or study period.		
**14b) CONSORT: Why the trial ended or was stopped (early)**		
Yes. "From April 18 to May 24, 2023, 2,177 participants were assessed for eligibility." "The trial period was three months."		
**15) CONSORT: A table showing baseline demographic and clinical characteristics for each group**		
"Results from the Baseline Demographics Questionnaire are given below in Tables 1 and 2."		
**15-i) Report demographics associated with digital divide issues**		
"Participants in both arms had similar mean ages and comparable marital statuses. Participants with a bachelor’s degree were the most represented overall, with 53% (50/95) in the intervention arm and 48% (22/46) in the control arm. Parity was not significantly different in either arm."		
**16a) CONSORT: For each group, number of participants (denominator) included in each analysis and whether the analysis was by original assigned groups**		
**16-i) Report multiple “denominators” and provide definitions**		
"Among participants in the intervention arm, 82/95 (86.3%) demonstrated an EPDS improvement of ≥4 points, compared to 11/46 (23.9%) in the control arm (p<.0001)."<br><br>"Responder analysis for the FAS population was performed on 130 participants (Table 4). Results show that, among intervention arm participants (n=90), 82/90 (91.1%) demonstrated an EPDS improvement of ≥4 points, compared to only 11/40 (27.5%) in the sham control arm (p<.0001)."		
**16-ii) Primary analysis should be intent-to-treat**		
Yes. "Analysis for the ITT population was performed on 141 participants, including 95 in the intervention arm and 46 in the control arm (Table 3). Among participants in the intervention arm, 82/95 (86.3%) demonstrated an EPDS improvement of ≥4 points, compared to 11/46 (23.9%) in the control arm (p<. 0001)."		
**17a) CONSORT: For each primary and secondary outcome, results for each group, and the estimated effect size and its precision (such as 95% confidence interval)**		
Yes. Results for the primary: "Among participants in the intervention arm, 82/95 (86.3%) demonstrated an EPDS improvement of ≥4 points, compared to 11/46 (23.9%) in the control arm (p<.0001)."<br><br>Results for the secondary: "Among participants in the MamaLift Plus arm, 79/95 (83.2%) also met the secondary endpoint of demonstrating an improvement to <13 EPDS (p<.0001). This is compared to only 15 (32.6%) participants in the sham control arm who met the same endpoint."		
**17a-i) Presentation of process outcomes such as metrics of use and intensity of use**		
Use/engagement was measured according to completion of treatment modules and post-baseline assessments. The manuscript defines two thresholds of use: "The Intention to Treat (ITT) population included all randomized participants who started at least one module. The Full Analysis Set (FAS) is a subset of the ITT and included all participants randomized in the study who started at least 1 module and provided a post-baseline (either Week 4 or end of treatment) EPDS assessment."		
**17b) CONSORT: For binary outcomes, presentation of both absolute and relative effect sizes is recommended**		
"Among participants in the intervention arm, 82/95 (86.3%) demonstrated an EPDS improvement of ≥4 points, compared to 11/46 (23.9%) in the control arm (p<.0001)." Relative effect sizes can be derived based on this information.		
**18) CONSORT: Results of any other analyses performed, including subgroup analyses and adjusted analyses, distinguishing pre-specified from exploratory**		
Yes. Participants were stratified based on "new mom" status, and results are described with the following: "51 of the 60 new moms (85%) in the MamaLift Plus arm demonstrated an improvement of ≥4 points, compared to only 25.8% (8/31) of new moms in the sham control arm. Thus, efficacy results were similar for “New Mom = Yes” and “New Mom = No.”		
**18-i) Subgroup analysis of comparing only users**		
To elaborate, primary efficacy analyses and subgroup analyses are given for the ITT population, which is defined in the manuscript as the following: "The Intention to Treat (ITT) population included all randomized participants who started at least one module."		
**19) CONSORT: All important harms or unintended effects in each group**		
Yes, the manuscript addresses this subitem. The relevant text is the following: "A total of four AEs were identified, triaged, and documented in the study. Two were in the MamaLift Plus arm and two were in the sham control arm."		
**19-i) Include privacy breaches, technical problems**		
There were no privacy breaches or technical problems.		
**19-ii) Include qualitative feedback from participants or observations from staff/researchers**		
Qualitative feedback was not collected from participants in this study. However, extensive feedback was collected in a previous human factors trial, which is referenced in the manuscript.		
DISCUSSION		
**20) CONSORT: Trial limitations, addressing sources of potential bias, imprecision, multiplicity of analyses**		
**20-i) Typical limitations in ehealth trials**		
Yes. The relevant text is the following: "The main limitation of this study is that it does not provide insight into the long-term effect after the eight-week intervention is over."		
**21) CONSORT: Generalisability (external validity, applicability) of the trial findings**		
**21-i) Generalizability to other populations**		
Yes. Generalizability to a general patient population is discussed.		
**21-ii) Discuss if there were elements in the RCT that would be different in a routine application setting**		
Yes. The relevant text is the following: "Additionally, by the nature of the trial being decentralized, we may anticipate patients receiving MamaLift Plus in a clinical setting behaving differently than the ones in the SuMMER study."		
**22) CONSORT: Interpretation consistent with results, balancing benefits and harms, and considering other relevant evidence**		
**22-i) Restate study questions and summarize the answers suggested by the data, starting with primary outcomes and process outcomes (use)**		
"The present study evaluated MamaLift Plus’s efficacy in reducing symptoms of depression in women diagnosed with postpartum depression." "Responder analysis showed statistically significant and clinically meaningful differences between MamaLift Plus participants and sham control arm participants. In the analysis of the ITT population, 86.3% (82/95) of MamaLift Plus arm participants achieved an improvement of ≥4 points, compared to only 23.9% (11/46) in the sham control arm."		
**22-ii) Highlight unanswered new questions, suggest future research**		
"Future studies may evaluate the effectiveness of MamaLift Plus in higher-risk participants or in participants receiving care at an OBGYN practice."		
Other information		
**23) CONSORT: Registration number and name of trial registry**		

Yes. The manuscript provides the registration number and name of the trial registry. The relevant text is the following: "CT.gov identifier: NCT05958095)"		
**24) CONSORT: Where the full trial protocol can be accessed, if available**		
The trial protocol is not available at this time.		
**25) CONSORT: Sources of funding and other support (such as supply of drugs), role of funders**		
Yes. Sources of funding are discussed in the manuscript. The relevant text is the following: "Curio Digital Therapeutics developed MamaLift Plus and funded this investigation."		
**X26-i) Comment on ethics committee approval**		
"This study protocol received approval from Brany IRB prior to data collection."		
**x26-ii) Outline informed consent procedures**		
Methods for collecting informed consent and information provided in informed consent is described in the manuscript with the following text: "All participants provided informed consent online. The informed consent included information about the purpose, voluntary participation, withdrawal conditions, length of participation, key study procedures, payment terms, risks/benefits, alternatives to study participation, costs, and confidentiality. The informed consent also included contact information for study personnel and information about free federal mental health resources, including national mental health hotlines."		
**X26-iii) Safety and security procedures**		
"free federal mental health resources, including national mental health hotlines."		
**X27-i) State the relation of the study team towards the system being evaluated**		
Yes. MamaLift Plus was developed by Curio Digital Therapeutics.		
